# Extensive Skeletal Muscle Metastases in Malignant Pleural Mesothelioma Detected by FDG PET/CT

**DOI:** 10.1055/s-0043-1774730

**Published:** 2023-12-04

**Authors:** Mustafa Yilmaz, Ozan Kandemir, Ediz Tutar

**Affiliations:** 1 Department of Nuclear Medicine, Mugla Sitki Kocman University School of Medicine, Mugla, Turkey; 2 Ozel Biyotip Pathology Laboratory, Bursa, Turkey

**Keywords:** malignant pleural mesothelioma, skeletal muscle metastasis, FDG PET/CT, contrast-enhanced CT

## Abstract

Malignant pleural mesothelioma (MPM) is a rare but aggressive tumor originating from pleural mesothelial cells. Distant skeletal muscle metastasis is rare in MPM. A 54-year-old woman was diagnosed with epithelioid MPM and treated with surgery, chemotherapy, and radiotherapy 2 years ago. During follow-up, diffuse irregular pleural thickening with focal chest wall invasion in the right hemithorax and two small pleural thickenings in the left hemithorax were seen on control diagnostic contrast-enhanced computed tomography (CECT). Fluorine-18 fluorodeoxyglucose positron emission tomography/CT (FDG PET/CT) imaging was performed as part of restaging. PET showed diffusely increased FDG uptake in the recurrent right pleural tumor, and two hypermetabolic small metastatic foci in the contralateral pleura. In addition, multiple hypermetabolic areas of various sizes in various skeletal muscle localizations, suggestive of extensive muscle metastases were noted. Histopathologic study confirmed metastatic epithelioid MPM. FDG PET/CT revealed multiple muscle metastases which were not observed on earlier CECT and contributed to the visualization of more extensive metastatic involvements in the presented case with MPM. FDG PET/CT can detect rarely seen skeletal muscle metastases that are not visualized on diagnostic CT, and provides more accurate restaging of MPM.

## Introduction

Malignant pleural mesothelioma (MPM) is an aggressive form of malignancy with a poor prognosis of fewer than 12 months from the time of diagnosis. It originates from mesothelial cells forming the pleural lining. 1 It is strongly associated with remote asbestos exposure. Histopathologically, MPM has major three subgroups, epithelioid, the predominant subgroup, less commonly sarcomatoid and biphasic subtypes.

Dissemination of MPM is generally local and regional lymph nodes are the major metastatic sites of MPM. Distant metastases are uncommon. However, distant metastases from MPM are considered more common than previously reported and can involve the liver, spleen, lung, bone, adrenal, kidney, thyroid, and peritoneum, but more rarely the skeletal muscle and brain. 2 3 4 5

Fluorine-18 fluorodeoxyglucose positron emission tomography/computed tomography (FDG PET/CT) has been increasingly used for the presurgical characterization and staging, evaluation of the response to treatment, accurate detection of distant metastases, assessing recurrence, and selecting the site for tissue biopsy of MPM. 6 7 8

Here, we report a case of MPM with extensive muscle metastases of various sizes in various skeletal muscle localizations detected by FDG PET/CT but not seen on diagnostic contrast-enhanced CT (CECT).

## Case Report

**Fig. 1:**
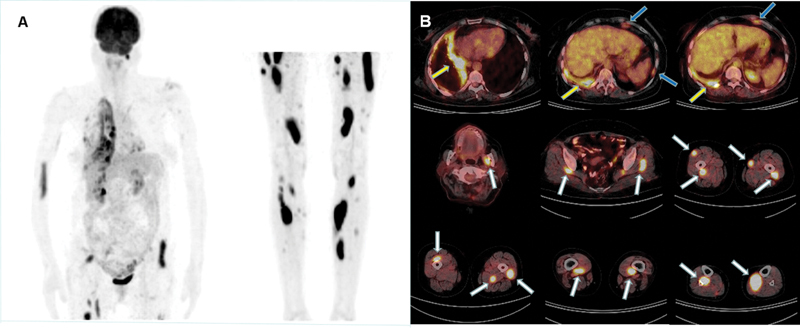
Increased fluorodeoxyglucose (FDG) uptake in the recurrent right pleural tumor ( **A** , maximum intensity projection [MIP] images; **B** , axial fused positron emission tomography/computed tomography [PET/CT] images, *yellow arrows* ), and two metastatic hypermetabolic small foci in the left pleura ( **B** , axial fused PET/CT images, *blue arrows* ). Multiple FDG avid foci of variable sizes in different skeletal muscle groups, including left medial pterygoid muscle, paraspinal muscles, bilateral gluteal muscles, right biceps brachii muscle, and extensively in the muscle groups of both lower extremities ( **A** , MIP images; **B** , axial fused PET/CT images, *white arrows* ).

**Fig. 2:**
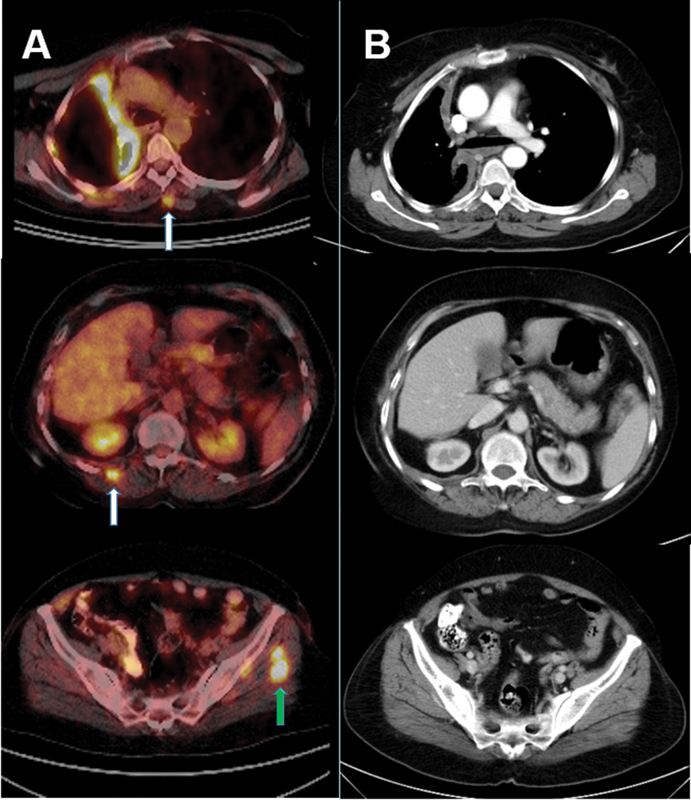
Fluorodeoxyglucose (FDG) avid metastatic lesions are clearly seen in the left and right paraspinal muscles ( **A** , fused positron emission tomography/computed tomography [PET/CT] images, *white arrows* ) and left gluteus medius muscle ( **A** , fused PET/CT image, *green arrow* ). No contrast-enhanced lesions are observed in the localizations corresponding to the hypermetabolic muscle lesions and muscle density of the metastatic foci is similar to normal muscles on contrast-enhanced computed tomography images, i.e., they are isodense muscle metastases ( **B** ).

A 54-year-old woman was diagnosed with epithelioid MPM 2 years ago and she was treated with right extrapleural pneumonectomy, hyperthermic intrathoracic chemotherapy, and radiotherapy. Because of her right-sided chest pain complaint, a control diagnostic CT scan of the chest, abdomen, and pelvis was obtained. Diffuse irregular pleural thickening with a focal chest wall invasion in the right hemithorax and two small pleural thickenings in the left hemithorax were seen on CT images. CT of the abdomen and pelvis did not show any abnormalities. Two weeks later FDG PET/CT imaging was performed as part of restaging. PET showed diffuse irregular high FDG uptake in the recurrent right pleural tumor, more prominent in the mediastinal side with maximum standardized uptake value (SUVmax) of 13.4, and also two hypermetabolic small foci in the left pleura, which suggest metastatic disease to the contralateral pleura (SUVmax: 2.7). In addition, PET/CT demonstrated multiple round, ovoid, or elongated hypermetabolic areas of various sizes in various skeletal muscle localizations, strikingly much more extensive in both legs, including the left medial pterygoid muscle, paraspinal muscles, both gluteal muscles, right biceps brachii muscle, and numerous muscles of both thighs and calves (SUVmax measured between 3.4 and 18.5) ( Fig. 1 ). On physical examination, the patient had only a slight swelling with mild pain in the lower part of the right biceps brachii muscle. Otherwise, she did not have any abnormal findings related to the skeletal muscles. After the FDG PET/CT findings, the CECT scan of the chest, abdomen, and pelvis was reviewed again, but no contrast-enhanced lesions were seen in the localizations corresponding to the hypermetabolic muscle lesions ( Fig. 2 ).

**Fig. 3:**
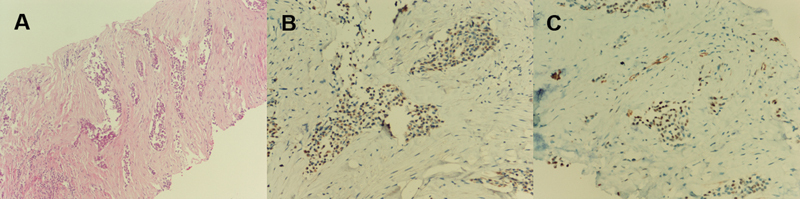
Histopathology of a hypermetabolic lesion biopsied from the left calf confirmed metastatic epithelioid malignant pleural mesothelioma (MPM). Invasion of the epithelioid neoplastic cells to the muscle tissue ( **A** , hematoxylin and eosin, ×100). Immunohistochemistry demonstrated positive staining for CK 5/6 ( **B** , ×200) and WT-1 ( **C** , ×200).

Biopsy from a relatively greater hypermetabolic muscle lesion located in the left calf and histopathology confirmed metastatic epithelioid MPM. Histopathologic examination demonstrated that invasion of the epithelioid neoplastic cells to the muscle tissue was present. The neoplastic cells stained positively for CK 5/6, WT-1, CK7, and CK20. Staining was negative for calretinin, thyroid transcription factor-1, carcinoembryonic antigen, and CDX-2 in the immunohistochemical study ( Fig. 3 ).

In addition to local recurrence and contralateral pleural metastases, FDG PET/CT was able to detect multiple muscle metastases, which were not observed on diagnostic CECT, and revealed more extensive metastatic involvements of the skeletal muscles in the presented case with MPM.

## Discussion

MPM is a rare cancer with increasing incidence and poor prognosis. 9 Presentation with metastasis is rare and cases with distant metastases are diagnosed more frequently during the clinical course of the disease. 10 Local metastatic spread is the major dissemination pattern. Distant dissemination is uncommon. However, distant metastases from MPM are considered more common than previously reported and can involve mostly the liver, spleen, lung, bone, adrenal, kidney, thyroid, and peritoneum. 2 3 Metastatic involvement of the skeletal muscle is rare among distant organ metastases in MPM. Not many but several MPM cases with distant muscle metastases have been reported in the literature. 4 5 6 11 12 13 14 15 16 17 It was also noted that the most common histological type of tumor in those cases was epithelioid MPM as observed in our patient.

FDG PET/CT imaging has been increasingly used for presurgical characterization and staging at presentation, for evaluation of response to treatment, and restaging during follow-up of patients with MPM. 6 7 8 Since FDG is a glucose analog, increased glucose use by malignant cells leads to high FDG uptake. Functional PET data, that is, increased FDG uptakes in malignant cells, along with anatomic CT data make FDG PET/CT a very useful and valuable imaging modality in most of malignant diseases.

Multiple skeletal muscle metastases from MPM detected by FDG PET/CT was first reported by Aukema et al. 12 FDG PET/CT was highlighted to be a superior and more effective imaging modality than CT alone in the evaluation of distant metastatic disease of MPM. 2 3 6 12 In the presented case, in addition to local recurrence and contralateral pleural metastases, PET/CT demonstrated extensive skeletal muscle metastases not suspected before, but there were no morphologic abnormalities on diagnostic CT corresponding to the hypermetabolic muscle lesions. As a result, FDG PET/CT provided more accurate restaging of our case with MPM.

The presence of skeletal muscle metastases are usually a rare entity with most malignancies; however, lung cancer has been reported as one of the most frequent causes of muscle metastases. 18 19 Due to the increasing use of hybrid imaging using FDG PET with CT in recent times, an increase in the frequency of detection of skeletal muscle metastases has been observed. 20 The exact underlying causes are not known yet, but there are some hypotheses as to why metastatic involvement in muscles is so rare. Muscle motion and mechanical tumor destruction, inhospitable muscle pH, and the ability of the muscle to remove tumor-produced lactic acid that induces tumor neovascularization are considered to act as defensive factors against the spread of the tumor. 21 22

## Conclusion

Rarely seen muscle metastasis in MPM may be missed on diagnostic CECT but FDG PET/CT can detect unusual or unexpected distant metastases such as skeletal muscle metastases and provides more accurate restaging of patients with MPM.

## References

[1] Cao C Tian D Park J Allan J Pataky K A Yan T D A systematic review and meta-analysis of surgical treatments for malignant pleural mesothelioma Lung Cancer 2014 83 02 240 245 24360321 10.1016/j.lungcan.2013.11.026

[2] Finn R S Brims F JH Gandhi A Postmortem findings of malignant pleural mesothelioma: a two-center study of 318 patients Chest 2012 142 05 1267 1273 22576637 10.1378/chest.11-3204

[3] Collins D C Sundar R Constantinidou A Radiological evaluation of malignant pleural mesothelioma - defining distant metastatic disease BMC Cancer 2020 20 01 1210 1216 33298007 10.1186/s12885-020-07662-y PMC7724793

[4] Grellner W Staak M Multiple skeletal muscle metastases from malignant pleural mesothelioma Pathol Res Pract 1995 191 05 456 460, discussion 461–462 7479364 10.1016/S0344-0338(11)80732-6

[5] Akyurek S Nalca Andrieu M Hicsonmez A Dizbay Sak S Kurtman C Skeletal muscle metastasis from malignant pleural mesothelioma Clin Oncol (R Coll Radiol) 2004 16 08 585 10.1016/j.clon.2004.09.004 15630859

[6] Zhang Y Edwards J Williams H Hao Z Khleif S Pucar D Unusual contiguous soft tissue spread of advanced malignant mesothelioma detected by FDG PET/CT Nucl Med Mol Imaging 2017 51 02 178 181 28559943 10.1007/s13139-016-0425-x PMC5429295

[7] Kitajima K Hashimoto M Katsuura T Clinical utility of FDG-PET/CT for post-surgery surveillance of malignant pleural mesothelioma - comparison with contrast-enhanced CT Oncotarget 2019 10 63 6816 6828 31827724 10.18632/oncotarget.27324 PMC6887579

[8] Truong M T Marom E M Erasmus J J Preoperative evaluation of patients with malignant pleural mesothelioma: role of integrated CT-PET imaging J Thorac Imaging 2006 21 02 146 153 16770231 10.1097/00005382-200605000-00006

[9] Robinson B M Malignant pleural mesothelioma: an epidemiological perspective Ann Cardiothorac Surg 2012 1 04 491 496 23977542 10.3978/j.issn.2225-319X.2012.11.04 PMC3741803

[10] Sibio S Sammartino P Accarpio F Metastasis of pleural mesothelioma presenting as bleeding colonic polyp Ann Thorac Surg 2011 92 05 1898 1901 22051293 10.1016/j.athoracsur.2011.04.117

[11] Laurini J A Castiglioni T Elsner B Soft tissue metastasis as initial manifestation of pleural malignant mesothelioma: a case report Int J Surg Pathol 1999 7 39 44

[12] Aukema T S Teunissen J J Burgers S A van Pel R Vogel W V Extensive soft-tissue metastases from malignant pleural mesothelioma J Clin Oncol 2009 27 21 e24 e25 19506158 10.1200/JCO.2008.21.0351

[13] Tertemiz K C Ozgen Alpaydin A Gurel D Savas R Gulcu A Akkoclu A Multiple distant metastases in a case of malignant pleural mesothelioma Respir Med Case Rep 2014 13 16 18 26029551 10.1016/j.rmcr.2014.07.003 PMC4246255

[14] Chiang C C Hsieh M S Chang D Y Malignant pleural mesothelioma with extensive skeletal muscle metastasis J Cancer Res Pract 2014 1 02 134 139

[15] Moysset I Valderas G Losa F Malignant pleural mesothelioma with metastases in the abdomen and left buttock: a case report J Case Rep Images Pathol 2017 3 13 16

[16] Cruz Castellanos P González Merino T de Castro Carpeño J Afectacion muscular de un mesothelioma maligno pleural de larga evalucion/Muscle involvement in long-term malignant pleural mesothelioma (in English) Arch Bronconeumol (Engl Ed) 2018 54 05 284 285 29132764 10.1016/j.arbres.2017.08.018

[17] Sunitha S Shah A H Gami A Trivedi P Thigh mass in a patient with malignant pleural mesothelioma: metastasis at an unusual site Indian J Pathol Microbiol 2021 64 04 834 836 34673618 10.4103/IJPM.IJPM_463_20

[18] Khandelwal A R Takalkar A M Lilien D L Ravi A Skeletal muscle metastases on FDG PET/CT imaging Clin Nucl Med 2012 37 06 575 579 22614189 10.1097/RLU.0b013e3182443e32

[19] Lupi A Weber M Del Fiore P The role of radiological and hybrid imaging for muscle metastases: a systematic review Eur Radiol 2020 30 04 2209 2219 31834507 10.1007/s00330-019-06555-4

[20] Haygood T M Wong J Lin J C Skeletal muscle metastases: a three-part study of a not-so-rare entity Skeletal Radiol 2012 41 08 899 909 22101865 10.1007/s00256-011-1319-8

[21] Pretorius E S Fishman E K Helical CT of skeletal muscle metastases from primary carcinomas AJR Am J Roentgenol 2000 174 02 401 404 10658714 10.2214/ajr.174.2.1740401

[22] Baser S Fisekci F E Bir F Karabulut N Rhomboideus major muscle metastasis as an initial clinical manifestation of pulmonary adenocarcinoma Thorax 2004 59 08 728 15282400 10.1136/thx.2004.026815 PMC1747101

